# Unraveling the enigma: Post-translational modifications in psychiatric disorders and their regulatory mechanisms

**DOI:** 10.1515/jtim-2026-0033

**Published:** 2026-06-13

**Authors:** Ting Hu, Yan Liao, Chenwei Huang, Haoling Zhang, Yadong Guo, Wangzheqi Zhang, Chunlei Jiang

**Affiliations:** Department of Stress Medicine, Faculty of Psychology, Naval Medical University, Shanghai, China; Mental Health Center, No. 940 Hospital of the Joint Logistics Support Force, Lanzhou, Gansu Province, China; Department of Anesthesia, Naval Medical University, Shanghai, China; Department of Biomedical Sciences, Advanced Medical and Dental Institute, Universiti Sains Malaysia, Kepala Batas, Penang, Malaysia; Department of Urology, Shanghai Tenth People’s Hospital, Tongji University, Shanghai, China

**Keywords:** psychiatric disorders, post translational modification, neurotransmitter, neurodevelopment

## Abstract

Psychiatric disorders, including schizophrenia, bipolar disorder, and major depressive disorder, are a group of categorical syndromes characterized by significant impairment of an individual’s cognition, emotional regulation, or abnormal behavior, causing severe distress and impairment in social functioning. Post-translational modifications (PTMs), such as phosphorylation, ubiquitination, and acetylation, are fundamental regulatory mechanisms in the brain, influencing diverse processes ranging from neuronal differentiation, synaptic plasticity, and neurotransmission, shaping the intricate cellular dynamics of the central nervous system. Understanding PTMs regulation in psychiatric disorders unveils their crucial functions in shaping neural networks, impacting learning and memory through mechanisms like long-term potentiation and depression. Additionally, disruptions in PTMs patterns have been associated with psychiatric disorders, suggesting their potential as therapeutic targets. Considering the critical role of PTMs in various cellular processes and signaling pathways, they could be a breakthrough in understanding psychiatric disorders. A holistic exploration of the PTMs landscape holds transformative promise for comprehending and managing a spectrum of psychiatric disorders.

## Introduction

Psychiatric disorders (*e.g*., schizophrenia, bipolar disorder, major depressive disorder) are syndromes characterized by cognitive/ emotional/behavioral impairments and social dysfunction.^[1]^ Diagnosis relies on symptom clusters, with significant clinical heterogeneity and unclear etiology.^[2]^ Studies from monozygotic (genetically identical) twins and the high failure rates of treatment basing single-mechanism both underscore the concept that psychiatric disorders cannot be thoroughly explained by a singular mechanism.^[3]^ Emerging evidence highlights gene-environment interactions as central to pathogenesis, and as research deepens, from the imbalance of neurotransmitter system to the coordinated changes in multiple dimensions such as neuroinflammation are reshaping the understanding of the nature of these diseases.^[4,5]^ Clarifying the pathogenic mechanisms is of great significance for the diagnosis and treatment of psychiatric disorders. Therefore, it is crucial to adopt an integrative and interconnected approach to understanding these complex conditions.

Post-translational modifications (PTMs), including phosphorylation, ubiquitination, and acetylation, play crucial roles in modulating protein functions, localizations, and interactions. Over 300 PTMs types fine-tune protein function dynamically.^[6]^ In central nervous system, PTMs regulate critical cellular processes like neurotransmitter system and synaptic plasticity.^[7]^ For instance, dopamine transporter (DAT) is a key protein that affects dopamine reuptake and regulates dopamine signaling, thereby controlling neurofunctions such as reward and cognition. ^[8]^ The plasma membrane stability and internalization of DAT can be regulated by presynaptic proteins and other signaling systems. Interesting, dopamine D3 receptors can bidirectionally regulate DAT, and chronic activation can promote the phosphorylation and ubiquitination of DAT, leading to its degradation.^[9]^ PTMs are characterized by their diversity and dynamics, and it is shown that key pathological molecules involved in the development of psychiatric disorders undergo various types of PTMs dysregulation, suggesting their roles in neural network shaping and disease pathogenesis.^[10]^

Overall, the current explanation of PTMs in the context of psychiatric disorders is still not in-depth enough. Considering the crucial role of PTMs in various cellular processes and signaling pathways, they may serve as a breakthrough for understanding psychiatric illnesses. This review synthesizes PTMs-mediated regulatory mechanisms in psychiatric disorders, emphasizing their potential as therapeutic targets and keys to unraveling disease complexity.

## PTMs crosstalk increases the intricacy in response to environmental factors

PTMs serve as molecular switches that alter protein behavior, influencing everything from enzyme activity and protein stability to localization and interaction networks. ^[11]^ These modifications occur on histones and non-histone proteins, dynamically maintaining brain homeostasis to enable rapid responses to internal/ external stimuli. Most prevalent PTMs include phosphorylation, ubiquitination, methylation, acetylation, glycosylation, and S-palmitoylation which are mainly catalyzed by various enzymes or driven by no-enzymatic chemical reactions.^[12,13]^ Different types of PTMs and their effects on protein structure are illustrated in Table 1. PTMs crosstalk can occur at the same residue or adjacent and distal sites on the same protein sequence (*e.g*., phosphorylation at distinct sites). Also, the crosstalk could happen between different PTMs types, which is commonly seen between multi-protein complexes and components of signaling pathways (*e.g*., acetylation-ubiquitination).^[14]^

**Table 1:** A comprehensive list of critical post-translational modifications

PTMs	Modification motif	Amino acid modification site	Modification enzyme
N-glycosylation	GlcNAc	Asn	Glycosyltransferase
O-glycosylation	GalNAc	Ser, Thr	
Prenylation	Isoprene	Cys	Isoprene transferases
S-palmitoylation	C16:0	Cys	PAT and APT
N-myristoylation	Myristoyl	Gly	NMT
Acetylation	CH3CO	Lys	HAT and HDAC
Phosphorylation	PO3^2–^	Ser, Thr, Tyr	Kinase and Phosphatase
Uniquitination	Ubiquitin	Lys	UB ligase and DUB
Methylation	CH3	Lys, Arg	methyltransferase and demethylase
S-Nitrosylation	Nitrogen monoxide	Cys	Nitric oxide synthase
SUMOylation	Small ubiquitin-like modifier	Lys	UB ligase and SENP
Glutathionylation	GSH	Cys	Glutaredoxin
Neddylation	NEDD8	Lys	NAE
Monoaminylation	Monoamine	Gln	TGM2
Lactylation	lysine	Lys	lactate transferase
Crotonylation	Crotonyl	Lys	Crotonyltransferase

PTMs: post-translational modifications.

Since protein activity largely depends on PTMs, PTMs combination provide pivotal but flexible regulation to cope with subtle changes in the environment. For example, the crosstalk of poly (ADP-ribose) polymerase 1 (PARP1) modification, deacetylation and ubiquitination, could be mobilized by Sirtuin 2 (SIRT2) to actively response to the stimuli of oxidative stress for relieving impairment.^[15]^ Neural plasticity is the core mechanism by which the brain adapts to external stimuli and plays a key role in the occurrence and development of neuropsychiatric diseases. The postsynaptic density (PSD) located beneath the postsynaptic membrane at dendritic spine tips is an electron-dense protein with assembly proteins such as adhesion proteins, scaffold proteins, and cytoskeletal proteins.^[16]^ Neuronal homeostasis is maintained through synaptic plasticity, which orchestrates the dynamic turnover of PSD proteins to dynamically stabilize the strength of synaptic signaling. Guanylate kinase-associated protein (GKAP), a scaffold protein that bridges N-methyl-D-aspartic acid receptors (NMDAR) with PSD protein complexes, is precisely regulated by Calcium Calmodulin Dependent Protein Kinase II (CaMKII)-mediated PTMs.^[17]^ Specifically, NMDAR activation triggers CaMKII phosphorylation of GKAP at serine-54, leading to its polyubiquitination and subsequent degradation, thereby removing GKAP from the synapse. Conversely, under conditions of reduced synaptic activity, phosphorylation of GKAP at serine-340 and serine-384 is enhanced, facilitating its recruitment to the synaptic site. Perturbations of protein modification in this process may be accompanied by synaptic loss of NMDAR, affecting synaptic transmission and ultimately leading to various neuropsychiatric disorders. Besides, there is also a cross-competition between glycosylation and phosphorylation at the Thr306 site of CaMKII itself, and the auto-phosphorylation of Thr305/Thr306 produces negative feedback to inhibit CaMKII overactivation.^[18]^

The diversity and interplay of PTMs are linked to key mechanisms involved in many psychiatric disorders, like neurodevelopment and pathological protein deposition.^[19]^ Tau protein is not only a signature pathological protein in neurodegenerative diseases such as Alzheimer’s disease (AD), but also plays a role in mental illness. Interestingly, hyperphosphorylated tau protein detected in the brain of AD patients leads to destabilization of microtubules and tangle of nerve fibers,^[20]^ but early phosphorylation of tau protein promotes its cumulative hyperubiquitination and accelerates its degradation.^[21]^ PTMs can modulate protein function in such a versatile manner, and the complexity is not just a biological curiosity but a fundamental aspect of how the brain encodes information, adapts to new experiences, and responds to pathological challenges.^[22,23]^ It is important to clarify how disrupted PTMs of key pathological molecules may lead to synapses, circuit defects, neurotransmitter disorders, and abnormal behavior. Understanding PTMs and its crosstalk, therefore, is crucial for decoding the brain’s molecular language and developing targeted therapies for a range of psychiatric diseases.

## PTMs in psychiatry from clinical and postmortem brain evidence

**Figure 1:**
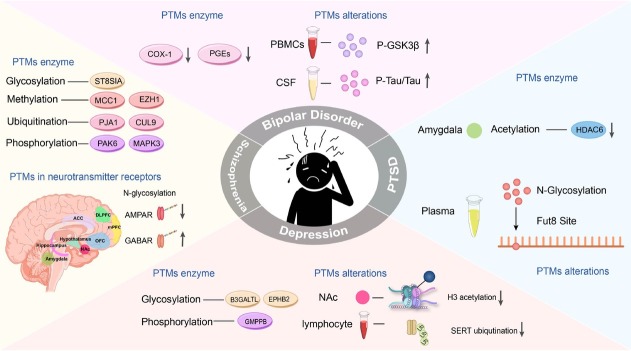
Overview of PTMs abnormalities associated with psychiatric disorders in clinic. Clinical evidence demonstrates that patients with schizophrenia, depression, bipolar disorder, and PTSD all exhibit altered PTMs profiles, albeit with varying sources of research evidence. Schizophrenia studies, primarily based on postmortem brain tissues, reveal abnormalities in phosphorylation, glycosylation, ubiquitination, and methylation of synaptic plasticity-related proteins across multiple brain regions such as the DLPFC and ACC, with key enzymes including methylation enzymes (MCC1 and EZH1), ubiquitination enzymes (PJA1 and CUL9) and phosphorylation enzymes (PAK6/MAPK3), among others. In bipolar disorder, PTMs anomalies are mainly characterized by altered expression of phosphorylation, showing different trends in GSK3β and Tau. Among them, the phosphorylation level of Tua protein was significantly increased in the CSF of BD patients. Investigations into depression and PTSD predominantly rely on peripheral blood (serum/plasma) assays, encompassing changes related to glycosylation and activity alterations of various glycosylation-modifying enzymes (*e.g*., GMPPB, FUT8). Overall, aside from schizophrenia, PTMs evidence derived from brain tissues remains scarce for other mental disorders, rendering peripheral blood detection the primary research methodology. PTSD: post-traumatic stress disorder; PTMs: post-translational modifications; DLPFC: dorsolateral prefrontal cortex; ACC: anterior cingulate cortex; CSF: cerebrospinal fluid; BD: bipolar disorder.

Genetic and environmental risk factors interact in complex ways to influence psychiatric disorders, yet the functional changes at the cellular level remain poorly understood. Proteomic analyses of blood and postmortem brain tissues have identified key PTMs such as phosphorylation, glycosylation, ubiquitination, and lipid modifications in psychiatric disorders (Table 2). Evidence of PTMs from clinical patients remains insufficient in psychiatric illnesses other than schizophrenia and mood disorders, and the relevant changes found in existing researches were presented in Figure 1.

**Table 2:** Summary of clinical and postmortem brain evidence for altered post-translational modifications in psychiatric disorders

PTM		Disease	Alterations	Method	Tissue category	Area	Reference
glycosylation	N-glycosylation	Schizophrenia	GluA2 subunit ↓	WB; ED and LBA	Post-mortem brain	DLPFC	Tucholski *et at*. (2013)^[33]^
	N-glycosylation	Schizophrenia	KA GluR6 subunit ↑	WB and ED	Post-mortem brain	DLPFC	Tucholski *et al*. (2013)^[34]^
	N-glycosylation	Schizophrenia	EAAT1/EAAT2 ↓	WB and ED	Post-mortem brain	ACC(EAAT1); DLPFC(EAAT2)	Bauer *et al*. (2010)^[37]^
	N-glycosylation	Schizophrenia	GABAAR subunits β2 ↑	WB; ED and LBA	Post-mortem brain	STG	Mueller *et al*. (2014)^[35]^
	N-glycosylation	Schizophrenia	GABAAR β2 subunit ↑	WB and ED	Post-mortem brain	STG subcellular ER; SYN	Mueller *et al*. (2015)^[36]^
	N-glycosylation	Schizophrenia	sialylated glycans ↓ in CSF; sialylated glycan ↑ in serum	-	CSF; Blood	Serum	Stanta *et al*. (2010)^[169]^
	O-glycosylation	Schizophrenia	O-GalNaAc ↓; O-GIcNAcylation ↑	WB	Post-mortem brain	STG	Mueller *et al*. (2018)^[170]^
	Glycosyltransferases	Schizophrenia	UGGT2 ↑	CO-IP; WB	Post-mortem brain	DLPFC	Kim *et al*. (2018)^[90]^
	Glycosyltransferases	Schizophrenia	B3GNT8 MGAT4A ↓	WB	Post-mortem brain	DLPFC	Kippe *et at*. (2015)^[91]^
	Glycosyltransferases	Schizophrenia	POFUT2 ↑; FUT8 ↓	WB	Post-mortem brain	STG	Mueller *et al*. (2017)^[92]^
	PSA-NCAM	Schizophrenia	cNCAM ↑	CO-IP; WB	CSF	-	Vawter *et al*. (2001)^[171]^
	PSA-NCAM	Schizophrenia; MDD; BD	PSA ↑ in SZ, not in BD or MDD	ELISA	Blood	Serum	Müller-Miny ^[164]^ *et al*. (2023)
	PSA-NCAM	Schizophrenia	NCAM ↓	ELISA	Blood	Serum	An *et al*. (2020)^[172]^
	N-glycosylation	PTSD	GlycoAge Test ↑; N-glycosylation paek change	DSA-FACE	Blood	Plasma	Moreno-Villanueva *et al*. (2013) ^[173]^
	N-glycosylation	PTSD	N-glycans (A2G2S1, A3G3S3, A4G4S4, A4F1G4S4 ↑); (FA2G2S1, M9 ↓)	HILIC	Blood	Serum	Tudor *et al*. (2019)^[174]^
	N-glycosylation	MDD	N-glycan peak (NG 1A2F ↓); (NA2, NA3(NA2, NA3FB ↑)	DSA-FACE	Blood	Serum	Boeck *et al*. (2018)^[51]^
	N-glycosylation	MDD	N-glycans peaks of plasma protein and IgG correlated with HDRS scores	HILIC-UPLC	Blood	Plasma	Park *et at*. (2018)^[52]^
	Glycosyltransferases	MDD	Sia-alpha2-6Gal/GalNAc ↓	qPCR; lectin micro-array	Blood	plasma; leukocytes	Yamagata *et al*. (2018) ^[93]^
lipid modification	Myristoylation	Schizophrenia	MARCKS; p-MARCKS ↓; myristoylation enzyme no difference	WB	Post-mortem brain	DLPFC	Pinner *et al*. (2014)^[175]^
	S-palmitoylation	Schizophrenia	protein S-palmitoylation ↓	mass spectrometry	Post-mortem brain	DLPFC	Pinner *et at*. (2016)^[43]^
	Prenyltransferase	Schizophrenia	FNTA; PGGT1B; RABGGTB ↓	WB	Post-mortem brain	DLPFC	Pinner *et al*. (2020)^[30]^
	Palmitoyl-thioesterase protein	Schizophrenia	PPT1 ↑	ELISA	Blood	Plasma	Wu *et at*. (2019)^[29]^
	Arachidonic Cascade Enzymes Acid	BD	COX-1; PGES (cPGES) ↓	WB	Post-mortem brain	Frontal cortex	Kim *et at*. (2011)^[176]^
Ubiquitination	ubiquitin	Schizophrenia	upregulated	ICC	Post-mortem brain	hippocampus	Nishimura ^[179]^ *et al* (2000)
	Ubiquitin; Ubiquitin enzyme	Scheizophrenia	ubiquitin ↓; K48 Polyubiquitination ↓; K63 Polyubiquitination ↑; UBA6 ↓; Nedd4 ↓; Ufl 1 ↓	WB	Post-mortem brain	STG	Rubio *et a!* (2013)^[178]^
	Ubiquitin; Polyubiq-uitination; Ubiquitin enzyme	Schizophrenia	ubiquitinated proteins ↑; blood ubiquitination enzyme ↓	WB	Post-mortem brain; Blood	OFC; Erythrocytes	Bousman *et al* (2019) ^[179]^
	Ubiquitin enzyme	Schizophrenia	FBXL21 ↓; MDM2 ↓	WB	Post-mortem brain	DLPFC	Andrews *et al* (2017)^[94]^
	Ubiquitin enzyme	Schizophrenia	UBE2K ↑	WB	Post-mortem brain; Blood	OFC; Erythrocytes	Meiklejohn *et al* (2019) ^[47]^
	Ubiquitin enzyme	Schizophrenia	UBE2M ↓; UCHL1 ↓	mass spectrometry	Post-mortem brain	hippocampus	Schubert *et al* (2015)^[95]^
	Ubiquitin enzyme	Schizophrenia	UBE3B ↓	IHC	Post-mortem brain	DLPFC	Kohlbrenner ^[46]^ *et al* (2018)
	Ubiquitins enzyme	Schizophrenia	UCHL1 ↓	ELISA	Blood	Serum	Demirel *et al* (2017)^[96]^
	Ubiquitin enzyme	Schizophrenia	Parkin ↑	WB	Post-mortem brain	DLPFC	Pandya *et al* (2014)^[97]^
	Ubiquitination	MDD	Ubiquitinated SERT ↓	WB-IP	Blood	lymphocyte	Mouri *et al*. (2016)^[56]^
Phosphorylation	Phosphoproteomic	Schizophrenia	P-STEP61/STEP61 ↓	WB	Post-mortem brain	anterior cingulate	Xu *et al*. (2016)^[180]^
	Phosphoproteomic	Schizophrenia	alterations enriched in complement and the coagulation systems	mass spectrometry	Blood	Serum	Jaros *et al*. (2012)^[181]^
	Phosphoproteomic	Schizophrenia	alterations enriched in signaling pathways	mass spectrometry	Post-mortem brain	corpus callosum	Saia-Cereda *et al*. (2016) ^[42]^
	Phosphorylation	BD	p-Tau/Tau ↑	ELISA	CSF	-	Jakobsson ^[69]^ *et al*. (2016)
	Phosphorylation enzyme	BD	p-state GSK3β ↑ in depressed or mixed	ELISA	Blood	PBMCs	Jacoby *et al*. (2016)^[182]^

"–": Data not reported or not applicable. PTSD: post-traumatic stress disorder; CSF: cerebrospinal fluid; ELISA: enzyme-linked immunosorbent assay; WB: Western Blot; LBA: ligand binding assay; Co-IP: co-immunoprecipitation; DSA-FACE: DNA sequencer-assisted fluorophore-assisted carbohydrate electrophoresis; HILIC: hydrophilic interaction liquid chromatography; MDD: major depressive disorder; BD: bipolar disorder; IHC: immunohistochemistry; DLPFC: dorsolateral prefrontal cortex.

### PTMs and schizophrenia

The pathogenesis of schizophrenia involves a complex interplay of genetic, environmental, and epigenetic factors that contribute to its manifestation.^[24]^ Genome-wide association studies (GWASs) have identified many schizophrenia susceptibility loci in which disease-associated variants are analyzed as potential risk factors of disease.^[25]^ Larger scale study of blood genomic loci and the study on brain tissue validation implicated that some genes coding PTMs related enzymes, such as glycosylation enzyme *ST8SIA* and *FUT9*,^[26]^ histone methylases and demethylases *MLL1* and *EZH1*,^[27]^ E3 ubiquitin ligases *PJA1* and *CUL9*, as well as protein kinase *PAK6* and *MAPK3*,^[28]^ were identified as fine mapped candidates of schizophrenia. In addition to the prevalent modifications, common protein lipidation include prenylation, N-myristoylation, and S-palmitoylation were also found in the blood serum^[29]^ and dorsolateral prefrontal cortex (DLPFC)^[30]^ in schizophrenia. A single-nucleotide polymorphism (SNP) associated with schizophrenia is located in the *DHHC8* gene, which is responsible for encoding enzymes related to palmitoylation.^[31]^ Most the top-associated risk variants frequently reside in regulatory elements rather than within coding exons. These alleles can modulate transcription-factor binding or chromatin accessibility, thereby altering the abundance or domain architecture of the encoded enzymes. Consequently, even a single-nucleotide change can propagate into quantitative shifts in receptor PTMs patterns that ultimately influence disease risk.

Collectively, emerging evidence indicates that PTMs that perturb synaptic homeostasis occupy a substantial mechanistic niche in the pathogenesis of schizophrenia.^[32]^ Moreover, brain area-specific glycosylation-phenotypes of neurotransmitter receptors for excitatory signaling, underscoring the molecular heterogeneity underlying the disorder. In the DLPFC of individuals with schizophrenia, a salient alteration is a pronounced reduction in the N-glycosylation level of the GluA2 subunit of α-amino-3-hydroxy-5-methyl-4-isoxazolepropionic acid receptor (AMPAR), accompanied by a concomitant increase in N-glycosylation of the GluR6 subunit of kainate receptors.^[33,34]^ However, γ-Aminobutyric acid A receptor (GABA_A_R) subunits that mediate inhibitory neurotransmission exhibit elevated N-glycosylation in the superior temporal gyrus (STG) of schizophrenia patients.^[35,36]^ Besides, an abnormal decrease of N-glycosylation of excitatory amino acid transporters (EAAT)1/2 in schizophrenia anterior cingulate cortex (ACC) and DLPFC that suggests a decreased capacity for glutamate reuptake.^[37]^ N-linked glycosylation is initiated in the endoplasmic reticulum, where proteins can be exfoliated by glycosidases or add additional oligosaccharide by Golgi-resident glycosyltransferases. This dynamic modification governs protein trafficking quality-control, tunes ligand-binding affinity by allosteric or electrostatic effects, and protects extracellular receptor domains from proteolytic turnover, thereby dictating synaptic receptor density and signaling kinetics.^[38]^ For instance, inhibition of N-glycosylation at the N370 site of GluA2 reduced cell surface expression controlled by the transport of AMPAR subunits from the endoplasmic reticulum to the cell surface.^[39]^ The above studies indicate that glutamate receptor glycosylation is broadly altered across multiple brain regions in schizophrenia, yet the direction of change is subunit- and area-specific. To move from descriptive findings to mechanistic insight, future studies must integrate GWASs for glycosylation enzyme such as *ST8SIA* with high-resolution spatial glycoproteomes to pinpoint which enzyme drives the observed glyco-signature of different subunits in exactly subregions that exhibit the strongest clinical-pathological correlates.^[40]^

Proteomic profiling of post-mortem schizophrenia brain tissue has revealed robust, region-specific dysregulation of proteins that govern axon guidance and dendritic-spine morphogenesis, implying that altered cytoskeletal dynamics and impaired synaptic connectivity are important molecular correlates of the disorder.^[41]^ Phosphoproteomic dissection of the corpus callosum further identified a schizophrenia-associated cluster of differentially phosphorylated proteins that are significantly enriched in the ciliary neurotrophic factor (CNTF) signaling. This cytokine-driven pathway governs cell survival, maturation and axonal ensheathment, suggesting that aberrant CNTF-mediated phosphorylation events contribute to callosal disconnection in the disorder.^[42]^ Besides, decreased protein palmitoylation of presynaptic and myelin-related proteins was found in schizophrenia patients’ DLPFC, most prominently the vesicular glutamate transporter 1 (VGLUT1), the small GTPase Ras, and myelin basic protein (MBP).^[43]^ The ubiquitin–proteasome system (UPS) is a central determinant of synaptic protein degradation and is indispensable for long-term potentiation and associative learning. Defective substrate ubiquitination or aberrant proteasomal degradation may destabilize the synaptic proteome and thereby contribute to the pathophysiology of schizophrenia.^[44,45]^ However, current pathological research on ubiquitination and other PTMs remains rather limited, with most efforts remaining at the level of expression changes of modifying enzymes, such as E3 ligases and deubiquitinating.^[46,47]^ The future research requires the use of a full-proteome-range PTMs map to systematically depict the panoramic changes of modified substrates in schizophrenia, thereby directly associating enzyme-substrate functions with the molecular causal chain of synaptic plasticity disorders.

### PTMs and depression

Major depressive disorder (MDD) is one of the most common psychiatric disorders, characterized by low mood, loss of motivation, despair sense and anhedonia.^[48]^ Enzymes involved in the phosphorylation and glycosylation process have been identified by multiple studies as risk factors for depression, such as *GMPPB*, *B3GALTL* and *EPHB2*.^[49]^ In a proteomic analysis of a large postmortem cohort focus on ACC region, Scifo *et al*. found the down-regulated proteins associated with persistent MDD pathology include GABA glutamate receptor signaling proteins (mGluR1 and EAAT3).^[50]^ The results of clinical studies do not indicate specific substrate targets for PTMs rather than provide the assessment of the overall level of glycosylation.^[51,52]^ However, preclinical studies have suggested that glycation mediates alterations in the expression of GABA_A_ receptors in depression. Specifically, deficiency of glycosylated α-Dystroglycan leads to the lower surface expression of GABA_A_R γ2 subunit in hippocampus and further depressive behaviors.^[53]^ Besides, glycosylated cannabinoid receptor was increased in postmortem prefrontal cortex (PFC) of subjects with MDD and could be downregulated by antidepressant treatment.^[54]^

Protein dysregulation signatures align anatomically with depression-implicated circuitry, exhibiting significant alterations in the hippocampus and middle temporal gyrus.^[55]^ Meanwhile, serotonin transporter (SERT) in peripheral lymphocytes is related to central expression to a certain extent. It is found that the downregulation of SERT protein ubiquitination in lymphocytes of MDD patients leads to insufficient degradation of SERT by the proteasome, and the change is related to the efficacy of antidepressants.^[56]^ Therefore, the peripheral SERT ubiquitination dynamics not only reflect the molecular imbalance of monoamine homeostasis but can also serve as a potential biomarker for efficacy stratification and individualized administration in MDD.

Stress is the most common trigger for depression, and stressful events might lead to alterations in epigenetic modifications with the form of histone modification.^[57]^ Histone PTMs constitute a key epigenetic layer that reshapes chromatin architecture and ultimately influences gene expression. For example, enhancement of histone 3 lysine 4 trimethylation (H3K4me3) at the promoter region of synapsin 1 was found in the brain tissues of MDD patients who died from suicide,^[58]^ subsequently came with the overexpression of synapsin. Aberrant histone modification of toll-like receptor signaling was observed in blood mononuclear cells of MDD, with reduced H3K4me3 levels at the promoter regions of *TNFAIP3*, *TLR4* and *TNIP2*.^[59]^ Evidence for histone-acetylation alterations has also emerged in patients with MDD. For example, Robison *et al*. reported that, relative to antidepressant-naïve patients, individuals receiving chronic antidepressant treatment exhibited markedly lower H3 acetylation at the CaMKIIα promoter within the nucleus accumbens (NAc),^[60]^ implicating activity-dependent chromatin remodeling in therapeutic response. Besides, histone-modifying enzymes, histone deacetylases (HDACs) and NAD^+^-dependent class III histone deacetylases SIRT, have moved to the forefront of depression research.^[61,62]^ Existing studies have reported inconsistent expression levels of the HDACs family in different brain regions and peripheral blood.^[63]^ This heterogeneity may stem from the differences in cell type composition and detection methods during the medication history and disease course stages, suggesting an urgent need for single-cell resolution, multi-center study with unified quality control standards was conducted to clarify the spatiotemporal characteristics of histone-modifying enzymes expression in MDD and its feasibility as a biomarker.

### PTMs and bipolar disorder

Bipolar disorder (BD) as a progressive neuropsychiatric illness is characterized by episodic recurrences of manic/ hypomanic, mixed and depressive states.^[64]^ Dopaminergic disruption has been responsible for manic symptoms in BD, which may originate from aberrant PTMs of DAT causing increasing extracellular dopamine.^[65]^ The evidence of increased 4-hydroxynonenal (4-HNE) in ACC among BD patients may suggest the subsequent sulfhydryl modification of DAT.^[66]^ Moreover, cytoskeleton-related proteins play a significant role in the formation of neuronal migration networks and synaptic function, important in the mechanism of BD.^[67]^ Tobe *et al*. demonstrated that human induced-pluripotent stem cell (hiPSC)-derived neurons from BD patients, and BD post-mortem brain, present a higher level of the inactivated form of collapsing response mediator protein 2 (CRMP2), namely phospho-Thr514 CRMP2.^[68]^ As a microtubule-binding protein, Tau is subject to a complex array of PTMs that collectively dictate its subcellular localization and microtubule-binding affinity.^[20]^ An increased hyperphosphorylated Tau in the cerebrospinal fluid (CSF) of BD patients have also been reported,^[69]^ leading to disruption of tau microtubule-binding properties and formation of tau aggregates.

In the treatment of BD, lithium has been considered the first option among therapeutic agents for its longterm mood stabilization. Meanwhile, lithium could alter various intracellular signaling cascade *via* alterations to regulatory PTMs.^[70]^ Interestingly, lithium has shown to inhibit glycogen synthase kinase 3β (GSK3β), further altering the phosphorylation state of substrates,^[71]^ such as the phosphorylation of CRMP2 at Thr514 and Thr522 residues.^[68]^ In addition to GSK3β, evidence suggests that dysregulated phosphorylation of protein kinase C (PKC), cAMP responsive element binding protein (CREB), and clock proteins, could contribute to the pathophysiology of BD.^[70]^ Lithium remains the gold-standard mood stabilizer for BD, yet its response rate barely exceeds 30% in naturalistic cohorts.^[70]^ Because lithium directly remodels the PTMs landscape, inter-individual heterogeneity in these PTMs signatures is likely to account for much of its variable efficacy. Future precision-medicine trials should empirically evaluate and quantitatively defined PTMs profiles, exhibiting relationships with clinical phenotypes and treatment outcomes in BD, thereby establishing PTMs signatures as mechanistically grounded, trial-stratifiable endotypes.

### PTMs and other psychiatric disorders

The development of post-traumatic stress disorder (PTSD) involves complex interactions between genetic susceptibility and exposure to traumatic stress.^[72]^ GWASs revealed that diseases were associated with stress-related genes and the glycolytic processes were significantly correlated with clinical features of PTSD.^[73,74]^ Specifically, it has been reported that the polymorphisms in the FUT8-related locus were mediated by defects in glycosylation in PTSD patients’ plasma.^[75]^ Besides, traumatic experiences (especially childhood abuse and early adversity) are significantly associated with changes in the methylation status of the *NR3C1* promoter,^[76]^ which affects the negative feedback regulation of the hypothalamic-pituitary-adrenal (HPA) axis by altering the expression of glucocorticoid receptors (GR), leading to the continuous activation of the stress response system.^[77]^ Results from saliva and blood samples showed that increased *NR3C1* methylation was associated with a lower risk of PTSD in male.^[78,79]^ HDAC6 was found to show a significantly reduced expression pattern in the amygdala of patients with PTSD.^[80]^ Despite this, evidence on HDACs-mediated PTMs in regulating GR nuclear translocation is indeed seriously insufficient, which is of great significance for explaining GR hypersensitivity in PTSD patients.

Among the risk genes related to autism spectrum disorder (ASD), kinases, genes expressing phosphatases and ubiquitin ligases account for a large proportion.^[81]^ Among the ASD patients, there were significant changes in the peak density of H3K4Me3 at hundreds of gene loci in PFC neurons in postmortem brain tissue,^[6]^ and in addition, one study showed that 68% of ASD cases shared same acetyl group signature of a large number of cis-regulatory elements in the PFC and temporal lobe region.^[82]^ Evidently, empirical evidence for PTMs alterations remains conspicuously sparse across psychiatric diagnoses beyond schizophrenia and MDD.

**Figure 2:**
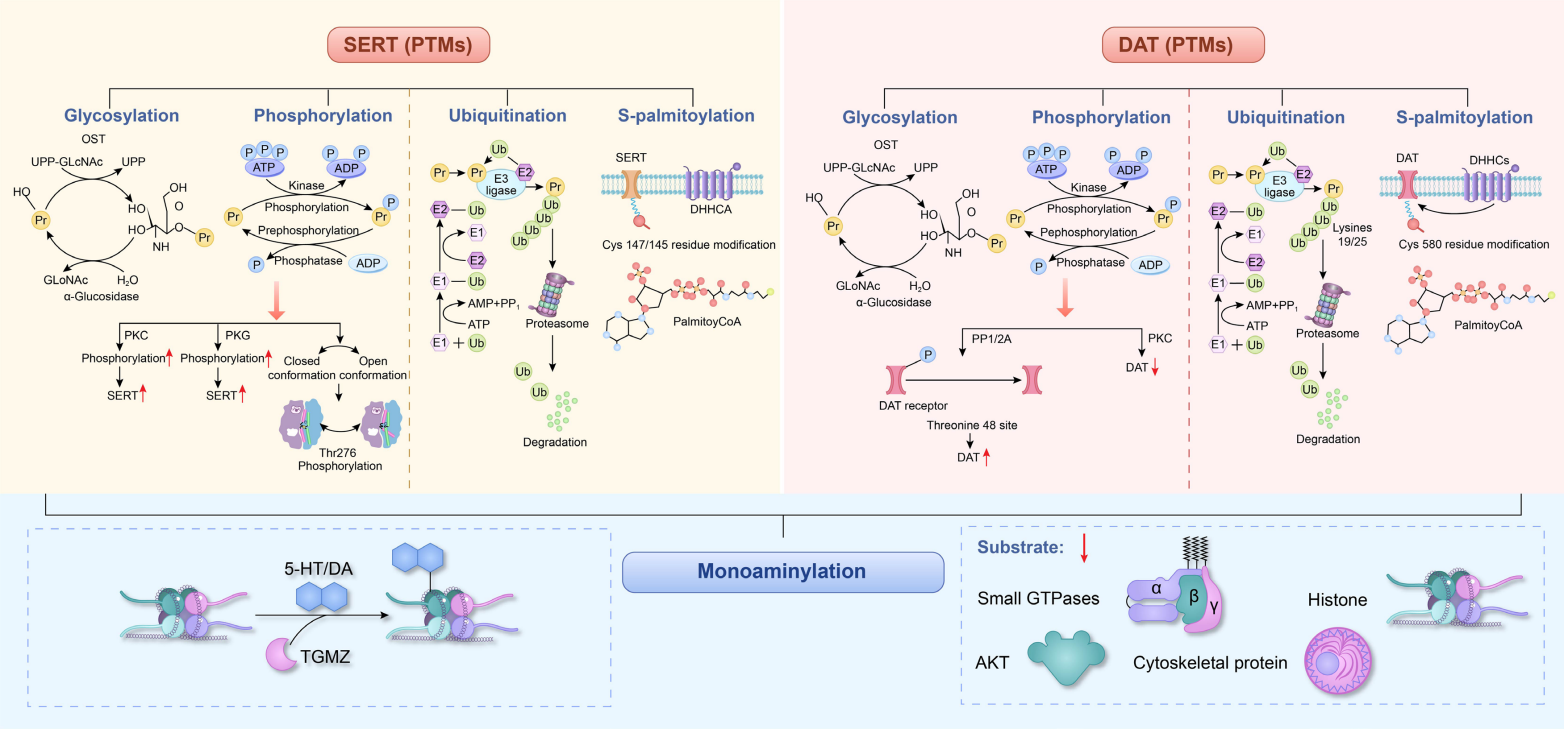
PTMs mechanisms of selective serotonin reuptake transporter and dopamine transporter. SERT and DAT are Na^+^/Cl^−^-dependent neurotransmitter transporters of the SLC6 family responsible for the reuptake of monoamine neurotransmitters in the synaptic cleft to presynaptic neurons. The enzyme OST (Oligosaccharyltransferase complex) and α-glucosidase can modify SERT and DAT. The resulting glycosylated products may influence the function and intracellular localization of these proteins. Under the catalysis of kinases PKC/PKG, SERT undergoes phosphorylation with increased active expression. Additionally, the transition to an open conformation can lead to the phosphorylation of SERT at the Threonine 276 site, thereby affecting the efficiency of reuptake function. However, turning the perspective to DAT is a different story. DAT is defused of phosphate groups under the action of phosphatase PP1 and PP2A, thereby enhancing its activity. E3 ubiquitin ligases can ubiquitinate transporter proteins, facilitating their degradation via the proteasome. Ubiquitination of DAT primarily occurs respectively at the lysine 19/25 sites accompanied with degradation. Moreover, the C-terminus of the SERT and DAT can both undergo S-palmitoylation modification through DHHC enzymes, regulating membrane binding and transporter activity. SERT and DAT directly affect the activation pattern of postsynaptic membrane receptors and regulate synaptic plasticity by controlling the time course of neurotransmitter action. Abnormal PTMs of monoamine transporter proteins leads to disturbances in signal conduction. On one hand, aberrant modifications such as excessive ubiquitination or glycosylation can mislead the degradation system with abnormal accumulation of the transporters, leading to neuronal toxicity and affecting individuals’ mood state and cognition function. On the other hand, deposition of monoamine neurotransmitters can facilitate the amination of substrates under the catalysis of transglutaminase 2 (TGM2), impacting the status of histones, small GTPases, the AKT pathway, and cytoskeletal proteins. These substrates further affect neuronal function through intracellular signal transduction systems. PTMs: post-translational modifications; SERT: serotonin reuptake transporter; DAT: dopamine transporter.

There is abundant evidence that several psychiatric illnesses, BD and schizophrenia as well, certainly constitute a spectrum of disorders, not a single entity.^[83,84]^ Overall, PTMs on neurotransmitter transporter receptor proteins are still the main pathological changes in psychiatric disorders.^[36,56]^ Focusing on dopamine and 5-hydroxytryptamine (5-HT) neurotransmitter transporters, this study discussed the existing reported PTMs and found that monoamine transmitters can further modify substrates through monoamination (Figure 2), among which substrate molecules such as histones and small GTPases also play an important role in various psychiatric disorders.^[85,86]^ Cross-disorder genetic studies have shown that multiple psychiatric disorders exhibit significant positive genetic correlations.^[10,87,88]^ Meanwhile, psychiatric disorders often share common risk factors and general psychopathology factors, such as stress.^[89]^ In this process, the key mechanism of the neurotransmitter hypothesis may connect various disorders and downstream pathological processes through the dynamic changes of PTMs. However, the heterogeneity among different disorders and within individuals of the same disorder reflects the biological complexity involved in the evolution of psychiatric disorders. For example, key modifying enzymes found in schizophrenia or depression, abnormal expression of glycosyltransferases and ubiquitin enzymes in different brain regions or peripheral blood,^[46,47,90-97]^ can help in the next step of research to determine possible substrates and cellular functions in different tissues for further precise treatment. In the process, PTMs may serve as the key to revealing the potential biomarkers for psychiatric comorbidity and enhance our understanding of personalized traits. Despite, samples from different brain regions and different protein substrates posed difficulties for the current horizontal comparison of diseases. In the future, it is necessary to carry out multi-center PTMs analysis of common targets for multiple diseases.

## Mechanisms of PTMs in psychiatry

The deformation or degradation of post-mortem brain tissue further increases the limitation in terms of differentiating primary and secondary pathology.^[98]^ Therefore, studying PTMs in preclinical models of psychiatry enable to further understanding the underlying mechanisms. The shared genetic basis, similarity in neurobiological findings, and overlapping clinical feature help to better construct the panorama of psychiatric disorders, while making it arduous to build animal models that encompass the complexity.^[99]^ Here, we aimed to dissect the major shared pathophysiological mechanisms in psychiatric disorders and analyze the potential roles of PTMs in established animal models.

### PTMs related to risk gene mutations

Genetic risk factors play a significant role in the pathogenesis of psychiatric disorders, a fact that has been fully confirmed through numerous family studies, twin heritability, and GWAS.^[100]^ Taking schizophrenia and bipolar disorder as examples, their twin heritability is as high as 81% and 85% respectively,^[101]^ indicate that genetic factors play a key role in disease susceptibility. These risk factors not only include rare chromosomal variations and copy number variations (CNVs), but also cover polygenic risk scores composed of common SNPs, which regulate neural development, synaptic plasticity and the function of the neurotransmitter system increase the risk of diseases. Although the pathogenic contribution of single-gene variations at the population level is limited, modeling these variants in preclinical models still provides irreplaceable experimental evidence for clarifying the pathological mechanism of psychiatric disorders and revealing therapeutic targets.

Thereinto, some proteins encoded by risk genes are critical targets for PTMs, while mutations in these genes can significantly alter protein regulation, leading to pathological states. Psychiatric disorders associated mutations can lead to the loss of PTMs target sites or abnormal expression of modifying enzymes, thereby impacting protein unction and cellular processes, which are reviewed in Table 3. The function of risk genes associated with PTMs primarily falls into the following categories: synaptic formation and neurodevelopmental proteins (*DISC1*, *BAI1*, *NRXN1*, and *MAGE-D1*),^[102,103]^ cytoskeletal and structural proteins (*NFL* and *KIF 17/3B* genes),^[104,105]^ signal transduction receptor proteins (*STEP*, *NRG1*, *GHR*),^[106,107]^ modifying enzyme proteins (*PCAF*, *USP9X*, *DGK*, *OGA*, *ZDHHC8/13*),^[109, 110]^ and transcriptional regulatory proteins (*BRD1*).^[111]^

**Table 3:** Risk gene mutation murine models and post-translational modifications of psychiatric disorders

Related diseases	Behavioral type	Knockout mice	Risk genes	Protein PTMs	Brain location	Downstream regulation	Reference
Schizophrenia	Damped circadian physiology and behaviors	Disc 1 knockout mice	DISC1	BMAL1 ubiquitination ↑	Hippocampus	-	Lee *et al*. (2021) ^[102]^
-	Deficits in spatial learning and memory	Bai 1 1 -/- mice	-	PSD-95 ubiquitination ↑	Hippocampus	decreased PSD thickness	Zhu *et al*. (2015) ^[183]^
Schizophrenia	Enhances mouse pup ultrasonic vocalization and reduces grooming	Nrxn 1ΔS5 mice	NRXN1	neurexin-1 heparan sulfate chains ↑	Hippocampus	increases excitatory synapse density	Lu *et al*. (2023) ^[103]^
Depression	Depression-like behavior including decrease in exploratory behavior, social interaction and sucrose preference, and increased immobility	MAGE-D1 knockout mice	-	SERT ubiquitination ↓	PFC	serotonergic signaling	Mouri *et al*. (2012)^[119]^
Schizophrenia	Deficit in social interaction and spatial memory	NFL +/- mice	NFL down	GluN1 ubiquitination ↑	Hippocampus	Reduced dendritic spine length and density and impaired CA1 LTP	Yuan *et al*. (2018)^[104]^
-	Memory Deficits	kif 17-/- Mice	-	NR2A Ubiquitination ↑	Hippocampus	impaired synaptic plasticity	Yin *et al*. (2011) ^[184]^
Schizophrenia	Lower social interaction and hyperactivity and anxiety-like behaviors	Kif3b +/- mice	Kif3b	CRMP2 carbonylation ↑	Hippocampus	Lamellipodial dynamic	Yoshihara *et al*. (2021)^[105]^ Carty *et al*.
Schizophrenia	Less sensitive to both the Locomotor and cognitive effects of phencyclidine treatment	STEP KO mice	STEP	p-ERK/ERK ↓	PFC	Increased PSD-95 levels	(2012)^[185]^
Schizophrenia	Increased locomotion and disrupted working memory and cognition	Nrg1 +/-mice; ErbB2/4 KO mice	NRG1; STEP61; ERBB4	STEP61 phosphorylation ↓ and ubiquitination ↓	PFC	decreases in phosphorylation of GluN2B and ERK1/2	Xu *et al*. (2018) ^[108]^
Anxiety	Increased anxiety-like behavior in elevated plus maze and light/dark (LD) box	Ghr KO mice	-	p-ERK/ERK ↑	Arcuate nucleus	-	Mahbod *et al*. (2018)^[107^]
ADHD/BD/schizophrenia	Hyperactivity	Usp9X -/Y mice	ANK3	ankyrin-G ubiquitination ↑	Forebrain	reduction of spine head size and density	Yoon *et al*. (2020)^[108]^
depression/OCD/autism	-	DGK62 knock-out mice	SERT	SERT ubiquitination ↓	-	-	Lu *et al*. (2020) ^[109]^
Depression	Short-term memory deficits and exaggerated response to acute stress	PCAF KO mice	-	p-ERK/ERK ↓	Hippocampus	decrease in the cell number in the pyramidal layer	Maurice *et al*. (2008)^[121]^
Depression	Antidepressant-like behavior	Oga +/- mice	lower O-GIcNAcylation	-	mPFC	reduced synaptic transmission	Cho *et al*. (2020) ^[110]^
Schizophrenia	-	22q11.2 deletion mouse model/ Zdhhc8 -/- mice	22q11.2 microdeletions/Zdhhc8	PSD95 S-palmitoylation ↓	Hippocampus	decreased density of dendritic spines and glutamatergic synapses	Mukai *et al*. (2008)^[186^
-	Increased sensorimotor gating, anxiety, hyperactivity, and decreased motor coordination	Zdhhc 13-/- Mice	Zdhc17	Drp 1 S-palmitoylation ↓	Cortex and cerebellum	Abnormal fission-fusion processes	Napoli *et al*. (2017)^[122]^
Schizophrenia	Reduced exploratory behavior and increased sensitivity to pentylenetetrazole-induced seizures	Brd 1^-/-^ mice	BRD1	histone H3 acetylation ↓	-	-	Paternoster *et al*. (2021)^[111]^

"–": Data not reported or not applicable. PTSD: post-traumatic stress disorder; PFC: prefrontal cortex.

Current research indicated that mouse models of schizophrenia primarily exhibit abnormal circadian rhythms, motor behavior abnormalities, social interaction deficits, and impaired cognitive function. Among these, gene knockout mice involving synaptic formation and neurodevelopmental proteins display significant behavioral abnormalities. For example, the disrupted in schizophrenia 1 (DISC1) gene, associated with neurodevelopment and synaptic function, is linked to schizophrenia and bipolar disorder. These genetic mutations increase susceptibility to psychiatric disorders by affecting neurodevelopment, synaptic plasticity, and neurotransmitter regulation.^[112]^ With increased ubiquitination of Basic Helix-Loop-Helix ARNT Like 1 (BMAL1), *DISC1* knockout mice exhibit suppressed circadian rhythms and behaviors through the inhibition of GSK3β.^[102]^ The elevation of ubiquitin-dependent degradation of GluN1 subunit of NMDAR could partially explain the deficit in social interaction and spatial memory.^[104]^ Synaptic properties like neurotransmitter release and receptor composition are crucial for brain function, which are regulated by transcellular complexes of synaptic organizing proteins. Among these, presynaptic neurexins are genetic risk factors for human brain-based disorders.^[113]^ The S5 insert of neurexins increases the number of heparan sulfate chains, which is associated with reduced neurexin- 1 protein level and reduced glutamatergic neurotransmitter release.

Depression rodent models show reduced sucrose preference and decreased social interaction, accompanied by short-term memory deficits and stress responses. Serotonin is a bioactive compound synthesized from tryptophan and is widely distributed throughout the brain, associated with emotional and motivational behaviors.^[114]^ Depressed patients showed reduced cerebrospinal fluid levels of 5-hydroxyindoleacetic acid (5-HIAA),^[115]^ and tryptophan depletion has been linked to transient relapses during successful treatment with selective serotonin reuptake inhibitors (SSRIs).^[116]^ Recent studies show that stabilizing the inward-open conformation of SERT increases its Thr276 phosphorylation, making the site accessible to kinases.^[117]^ The 5-HT uptake function relies on proper folding and cellular localization of SERT, regulated by PTMs like glycosylation, disulfide bond formation, and oligomerization.^[118]^ The knockout mice models of depression often involve abnormal expression of modifying enzyme genes. Melanoma-associated antigen D 1 (MAGE-D1) interacts with RING E3 ubiquitin ligases and is associated with protein degradation. Mouri *et al*. reported that MAGE-D1 knockout and knockdown in the PFC lead to depressive-like behaviors, likely due to MAGE-D1 binding to *via* the necdin homology domain, resulting in elevated SERT expression and reduced ubiquitination.^[119]^ Brain-specific diacylglycerol kinase δ (DGKδ) knockout mice displayed depressed and obsessive-compulsive disorder-like behaviors.^[120]^ Recent research showed that the phenotype was accompanied with significantly increased SERT protein levels. The catalytic subdomain-a and coiled-coil region of DGKδ interact with the C-terminal cytoplasmic region (CTC) of SERT. Additionally, DGKδ interacted with the MAGE-D1 adaptor protein and Praja-1 E3 ubiquitin ligase, facilitating SERT ubiquitination via Praja-1.^[109]^

In addition, risk genes associated with depressive phenotypes include *PCAF* and *OGA*, which are involved in histone acetylation and glycosylation modifications, respectively. Histone acetylation by proteins like CREB binding protein (CBP) and p300/CBP associated factor (PCAF) is crucial for brain plasticity and memory formation. Knockout studies reveal that *PCAF* deletion leads to memory deficits and exaggerated stress responses in mice.^[121]^ Glycation evidence has been reported in the peripheral blood and postmortem brains of patients with depression. UDP-GlcNAc acts as a donor for GlcNAc transfer to proteins composing the O-GlcNAcylation with highly dynamic and reversible forms. The processes of addition and removal of the GlcNAc moiety are exclusively mediated by O-GlcNAc transferase (OGT) and O-GlcNAcase (OGA), respectively. OGA heterozygous (Oga^+/−^) mice with chronically elevated O-GlcNAcylation levels showed reduce inhibitory synaptic transmission in mPFC and exhibited an antidepressant-like phenotype.^[109]^

Anxiety disorder models involve growth hormone receptor *GHR* knockout mice and Zdhhc13 knockout mice, with the latter also exhibiting hyperactive behaviors. Ghrelin, through its acylated and desacylated forms, influences anxiety and stress response. Lifelong ghrelin deficiency showed increased anxiety-like behavior.^[107]^ Mice carrying Zdhhc13 recessive mutation displayed increased sensorimotor gating, anxiety, hypoactivity, and decreased motor coordination. This behavioral abnormality may result from Zdhhc13-dependent dynamin-related protein 1 (DRP1) S-palmitoylation, leading to impaired mitochondrial fission-fusion processes. Consequently, this affects mitochondrial ATP output and impacts synaptic structure, neurotransmission, and integrity in the brain.^[122]^

While mouse models for bipolar disorder, attention deficit hyperactivity disorder (ADHD), and autism are not directly specified, it is evident that abnormal expression of many genes often points to multiple psychiatric phenotypes. For example, Yoon *et al*. demonstrated that the deubiquitination of proteins with ankyrin-repeat domains is crucial for the proper developmental trajectory of cortical synapses. Forebrain-specific *Usp9X* knockout mice with disruption of the deubiquitinase showed increase of ankyrin-G ubiquitination and clinical abnormalities like hyperactivity, a symptom included in ADHD and anxiety.^[108]^ However, due to limitations in behavioral measurements in animal models, pathological changes are frequently tightly linked to specific behavioral traits, and their clinical relevance remains at the stage of risk assessment. Many knockout mouse models exhibit abnormalities in specific brain regions, particularly the PFC^[106,110,119]^ and hippocampus,^[102, 103, 104, 105]^ which play key roles in cognitive function, emotion regulation, and synaptic plasticity, and their dysfunction is closely related to the pathological mechanisms of a variety of psychiatric disorders.

Mouse models of psychiatric disorders engineered to harbor risk genetic mutations predominantly manifest aberrant neurotransmitter release and plasticity impairments as critical intermediate pathways linking genetic vulnerability to behavioral phenotypes. Crucially, synaptic dysfunction in these models exhibits marked transdiagnostic convergence. For instance, whether it is the *DISC1* mutation related to schizophrenia or the *Oga* variation in depression, their synaptic phenotypes may eventually converge into behavioral characteristics shared by multiple diseases, such as impaired working memory, abnormal social behavior, or emotional regulation disorders.^[102,110]^ This multi-effect phenomenon of one cause suggests that synaptic pathology as a final common pathway across psychiatric diagnoses, thereby providing empirical support for dimensional frameworks of psychopathology that supersede traditional categorical classifications. On this basis, evaluating the biological basis of clinical symptoms by combining synaptic dysfunction in specific brain regions and the regulatory network of synaptic transmission-related proteins PTMs will promote the development of precision drugs based on symptom dimensions in the future.

### PTMs related to stress

The impact of the single gene on disease is still limited, but mutations with significant effects may sensitize individuals to exhibiting developmental neuropsychiatric disorders.^[101]^ The final behavioral disease phenotype of individuals still depends on environmental factors and the accumulation of polygenic risks. Stress as one of environmental factors plays an important role in the mechanism of psychiatric disorders.^[122]^ DSM-5 defines stressors as “any emotional, physical, social, economic or other factors that disrupt individuals’ physiological, cognitive, emotional or behavioral balance”.^[124]^ It is usually simply distinguished between physiological stress and social stress.^[125]^ Chronic stress is indeed a well-established factor in the development and exacerbation of various psychiatric disorders.^[123]^ It can lead to long-lasting changes in brain structure and function, contributing to the pathophysiology of these conditions. Environmental factors especially stress during critical periods of brain development, can also influence PTMs patterns, potentially triggering or exacerbating neuropsychiatric conditions (Table 4). This gene-environment interaction highlights the complexity of PTMs regulation for stress and its impact on mental health.

**Table 4:** Stress induced post-translational modifications in the psychiatric disorder-related models

Pathological process	Protein and Kia site alteration	Brain location	Cell location	Downstream regulation		Regulator	Intervention	Stress model	Reference
Neurotransmitter dys-regulation	HSP9O acetylation ↓	Dorsal raphe	Neuron	GR pathway in Serotonin Pathways		HDAC6	depletion of HDAC6	10-day CSDS	Espallergues *et al*. (2012)^[128]^
Neurotransmitter dys-regulation	GLT-1 ubiquitination ↓	PFC	-	Glutamate signaling		Ube2d2-Nedd4L	-	10-day CSDS	Kosuge *et al*. (2024) ^[132]^
Neurotransmitter dys-regulation	Csnk 1a1 phospholation ↑	mPFC; NAc	-	-		-	Ketamine	3-week CUMS	Xiao *et al*. (2020)^[160]^
Neurotransmitter dys-regulation	GLT-10-GIcNAcylation ↑	mPFC	Astrocytes	Glutamate signaling		OGT	Selective deletion of OGT in astrocytes	10-day CSDS and 3-day subthreshold SDC	Fan *et at*. (2023)^11311^
Synaptic plasiticity	GluN2B/USMG5/PSMA4 k63-polyubiquitin ↑	Amygdala	-	ATP synthesis		-	knockdown of K63-polyubiquitination	fear conditioning	Farrel *et al*. (2023) ^[139]^
Synaptic plasiticity	GluA1 Ser831 phospholation ↑; GSKβ Ser9 phospholation ↓	Hippocampus	-	mTORC1/4E-BP1 axis		-	fasudil	14-day CRS	Roman-Albasini *et al*. (2020)^[137]^
Synaptic plasiticity	Strn 4 S-palmitoylation ↑; Shank3, PSD-95 S-palmitoylation ↓	-	Neuron	-		-	-	21-day CRS	Zareba-Koziol *et al*. (2019)^[140]^
Synaptic plasiticity	global palmitoylation level, palmitoylation of PSD-95 and glutamate receptors ↑	Hippocampus	Neuron	Synaptic transmission strengthening		APT1	SiRNA-APT1	3-day Fear conditioning	Shen *et al*. (2022)^[141]^
Neurodevelopment	Nogo-A phospholation ↑	mPFC	-	RhoA/Rock1 pathway		KYNA	exercise	14-day CRS	Yan *et al*. (2024)^[187]^
Neurodevelopment	HIPK2 SUMOylation ↓	PFC	Neuron	Atoptosis signaling		-	Genipin 1-O-β-D-gentiobioside	4-week CUMS	Xia *et al*. (2024)^[188]^
Neuroinflammation	protein sulfenylation and nitrosylation ↑; ASK1 phospholation ↑	Hippocampus; frontal cortex	-	-		Thioredoxin	-	4-week CUMS	Zhou *et al*. (2019)^[189]^
Neuroinflammation	HSP9O k63 ubiquitination ↓	Hippocampus	Microglia	Microglia activation		HECTD1	Overexpression of circDYM	5-week CUS	Zhang *et al*. (2020) ^[190]^
Neuroinflammation	Myd88 ubiquitination ↓	Hippocampus	Microglia	NF-kB pathway		SPOP	Zuojinwan	7-week CUMS	Tao *et at*. (2023)^[145]^
Neuroinflammation	GR ubiquitination ↓	Hippocampus	-	Cytokine		PIAS1	overexpression of PIAS1	14-day CSDS and 3-day subthreshold SDC	Lin *et al*. (2020)^[147]^
Neuroinflammation	NLRP3 ubiquitination ↓	hippocampus	Microglia	Inflammation		MARCHF7	Saikosaponin-d	4-week CUMS	Gao *et at*. (2024)^[146]^
Epigenetic alterations	histone H3 lysine 27 mono-methylation ↑	NAc	Neuron	Neuronal excitability and neurotrans-mission		SUZ12	-	10-day CSDS, 3-day subthreshold social defeat and 7-day ELS	Torres-Berrio *et al*. (2024)^[152]^
Epigenetic alterations	histone H3 lysine 79 Dimethylation ↑	NAc	Neuron	Steroid hormone biosynthesis and metabolism		DOT1L and KDM2B	overexpression of Kdm2b	7-day ELS and 10-day CSDS	Kronman *et al*. (2021)^[154]^
Epigenetic alterations	histone H3 lysine 4 acetylation/trimethylation ↓	NAc	-	GDNF	HDAC2		imipramine	6-week CUMS	Uchida *et al*. (2011) ^[159]^
Epigenetic alterations	histone H3 acetylation ↑	NAc	Neuron	Rac1	HDAC		MS-275	10-day CSDS	Golden *et al*. (2013) ^[153]^
Epigenetic alterations	histone crotonylation ↓	mPFC	Neuron	Neuropeptide VGF nerve growth factor	CDYL		knockdown of CDYL	10-day CSDS and 4-week CUMS	Liu *et al*. (2019)^[191]^
Epigenetic alterations	histone H3 Iysine9 β-hydroxybutyrylation ↓	Hypothalamus	Neuron	-	β-hydroxybutyrate		β-hydroxybutyrate	3-week CRS	Chen *et at*. (2017)^[192]^
Epigenetic alterations	histone H1 lysine lactylation ↑	PFC	Neuron	-	Lactate		-	Electroconvulsive stimulation and 10-day CSDS	Hagihara *et al*. (2021)^[193]^
-	sialylated N-glycan ↑	Serum	-	-	-		-	3-week CUMS	Mahmoud *et al*. 2010^[194]^
Metabolism	ATP50 crotonylation ↓	Ovaries and plasma	-	Phospholipid metabolism	HDAC2		Increased HDAC2 Phosphorylation	Long term CRS	Chen *et al*. (2022)^[195]^

"–": Data not reported or not applicable. PFC: prefrontal cortex; NAc: nucleus accumbens; HDACs: Histone deacetylases; APT1: acyl-protein thioesterase 1; SPOP: speckle-type POZ protein; PIAS1: activated STAT1.

#### Molecular regulation of neurotransmitter signaling

The stress response first affects brain function through the release of neurotransmitters, among which PTMs are crucial determinants of neurotransmission, orchestrating the intricate signaling pathways that facilitate communication between neurons. Neurotransmitters, including dopamine, 5-HT, norepinephrine, and glutamate system, play a vital role in neural circuits signaling with precise and flexible modulation.^[126]^ While these neurotransmitters share overlapping projections and synaptic mechanisms, each is associated with distinct functions and psychiatric disorders. In chronic stress, glucocorticoid hormones induce GR nuclear translocation that leads to a downregulation of multiple serotonergic GR target genes including 5-HT_1A_.^[127]^ Cytoplasmic lysine deacetylase HDAC6, one of HDACs, could control heat shock protein 90 (HSP90) acetylation, and thereby modulate the HSP90–GR interaction and focal prevent the GR signaling in serotonin pathways.^[128]^

Besides, molecular dynamics simulations of the solute carrier family 6 (SLC6) family show that glycosylation does not significantly change the core structure of the transporter, but it modifies the dynamics of the extracellular loop and affects the conformational flexibility of the surrounding region.^[129]^ Changes in DAT glycosylation can lead to dysfunction in dopamine regulation, potentially causing anxiety/depressive-like behavioral changes.^[130]^ Besides, stress induced increase of glutamate transporter 1 (GLT-1) O-GlcNAcylation and impaired glutamate signaling in astrocytes.^[131]^ The ubiquitination at the cell surface involves internalization, endosomal sorting, and either recycling to the cell surface or targeting to the proteasome or lysosome for degradation, playing a role in regulating the activity of GLT-1. Chronic social defeat stress (CSDS) reduced depolarization-evoked glutamate release in PFC and caused a notable decrease of ubiquitinated GLT-1 in the PFC of CSDS-exposed mice.^[132]^ Furthermore, Nedd4L (an E3 ligase for GLT-1) downregulation may be involved in the decrease of GLT-1 ubiquitination, which may relate to stress-induced social impairment and depressive behaviors.^[132]^

#### Synaptic plasticity and neurodevelopment

Neurotransmitters have a profound impact on synaptic plasticity and neural development by regulating the intensity of synaptic transmission. Brain activity depends on the formation of functional networks of interconnected neurons, which involves the precise formation and maturation of synapses to establish efficient neuronal wiring in the developing brain.^[133]^ The intricate arrangement of protein networks on both sides of the synapse plays a crucial role in facilitating synaptic transmission and plasticity. Activity-dependent synaptic plasticity is a fundamental characteristic of the nervous system, enabling neurons to communicate and modify their connections based on previous experiences.^[134]^ Neuroplasticity and neurodevelopment have a profound impact on the manifestation and progression of clinical symptoms in many psychiatric illnesses, especially in the cognitive process.^[135]^

PTMs serve as fundamental regulators of neuroplasticity, influencing both long-term potentiation (LTP) and longterm depression (LTD), key mechanisms underlying learning and memory controlled by ionic glutamate receptors. Phosphorylation stands out among PTMs as it can rapidly modify neurotransmitter receptors and synaptic proteins. For instance, phosphorylation of glutamate receptors, such as AMPAR and NMDAR, modulates their trafficking, channel conductance, and responsiveness to ligands, thereby influencing synaptic strength and plasticity essential for learning and memory.^[136]^ Chronic stress reduced phosphorylation of ERK-2 and CREB, while increased synaptic GluA1 Ser831 phosphorylation in hippocampus. Administration of Rho-associated protein kinase inhibitor prevents both chronic stress-induced depressive-like behavior and synaptic PTMs alterations.^[137]^ UPS is also crucial for the degradation of ionic glutamate receptors. Recent studies have identified baseline and learning-related sex differences in UPS activity within brain regions essential for fear memory formation, particularly the amygdala.^[138]^ Farrell *et al*., found that K63-polyubiquitination is essential under fear conditioning stress in females by regulating ATP synthesis and proteasome activity following learning, such as GluN2B signaling.^[139]^ These modifications can dynamically alter the structural and functional properties of synaptic proteins, thereby fine-tuning synaptic strength in response to activity. Proteins, such as PSD-95, Striatin-4, CaMKII, and GSK3β that play key roles in regulating synaptic plasticity showed alterations in S- Palmitoylation/S-Nitrosylation crosstalk after chronic stress.^[140]^ Besides, under fear conditioning, global palmitoylation level, palmitoylation of PSD-95 and glutamate receptors were elevated in the hippocampus of mice with freezing behavior, which could be regulated by acyl-protein thioesterase 1 (APT1).^[141]^

For neurodevelopment, such as neuronal differentiation and synaptogenesis, PTMs act as precise molecular regulators that guide the fate of neural progenitor cells. Neurodevelopment necessitates the presence of dynamically adaptable microtubules. After chronic restrain stress (CRS), expression of tyrosinated alpha-tubulin, a primary component of microtubules, was significantly decreased in hippocampus while acetylated alpha-tubulin showed an increased trend after stress. Decrease in tyrosinated tubulin led to dendritic retraction and failure in neuronal plasticity.^[142]^ Similarly, study reported that rats exposed to chronic unpredictable mild stress (CUMS) showed impairment of microtubule dynamics accompanied with the decreased level of phosphor microtubule-associated proteins (MAP-2).^[143]^ The healthiness of the microtubule can determine the fate of neurons in many neurological disorders including depression-associated neurodegeneration.

#### Neuroinflammation

Neuroinflammation has been identified as a significant factor in the pathogenesis of psychiatric illnesses, causing a vicious cycle for different pathological processes.^[123]^ There is a complex interplay between different PTMs and neuroinflammatory processes.^[144]^

The degradation disturbance of ubiquitination has also been found in other neuroinflammatory pathways. CUMS stimulation resulted in decreased speckle-type POZ protein (SPOP) expression, impaired Myeloid differentiation primary response gene 88 (MyD88) ubiquitination, and activation of downstream nuclear factor kappa-B (NF-κB) signaling.^[145]^ Saikosaponin-d as a triterpene saponin from the roots of Bupleurum Chinese effectively alleviated depression-like symptoms induced by CUMS. It has antidepressant effects in CUMS mice by promoting ubiquitination of NOD-like receptor thermal protein domain associated protein 3 (NLRP3) to inhibit inflammasome activation and improve the inflammatory state.^[146]^

Moreover, protein and mRNA levels of small ubiquitin-like modifier (SUMO) E3 ligase protein inhibitor of activated STAT1 (PIAS1) were decreased in the hippocampus of high-susceptibility mice after CSDS. Local overexpression of PIAS1 in the hippocampus followed by CSDS exposure promoted stress resilience by attenuating social avoidance and improving anxiety-like behaviors. High-susceptibility mice displayed decreased levels of GR expression, and GR SUMOylation in the hippocampus was associated with stress vulnerability.^[147]^

#### Epigenetic alteration

Epigenetic alterations induced by histone modifications have been implicated in the pathogenesis of schizophrenia, depression, and bipolar disorder. Histone modifications play a crucial role in regulating gene expression by altering the accessibility of chromatin and thereby influencing the transcription of specific genes involved in neuronal function and synaptic plasticity.^[148]^ Acetylation of histone (*e.g*., H3K9ac, H3K27ac) can recruit bromine-containing transcription factors and chromatin remodeling complexes, while methylation (*e.g*., H3K4me3, H3K27me3) is recognized by effector proteins as “histone code”, so as to precisely regulate the spatiotemporal specific expression of neuronal plasticity genes such as *BDNF*, at the topological level of the three-dimensional genome.^[149,150]^

It is particularly noteworthy that HDACs, as key effector molecules of epigenetic regulatory networks, have made their bidirectional regulatory effects on stress susceptibility and antidepressant response an important pharmacological target for innovative intervention strategies for depression.^[151]^ HDACs are a family of lysine deacetylases that regulate protein function by removing acetyl groups from lysine residues. Recent studies indicate that broad pharmacological inhibition of class I and/ or class II HDACs can normalize behavioral deficits induced by CSDS.^[128]^ Stress-induced epigenetic changes may involve the histone modifications of genes such as Glial cell-derived neurotrophic factor (GDNF), Rac1 and genes related to neuronal excitability, which are closely associated with the onset and progression of depression.^[152,153]^

As an important distal risk factor for psychopathology in adulthood, early life stress (ELS) is mainly realized through histone modification during the critical period of development. Maternal separation, childhood abuse and other adversities can establish a persistent histone hypoacetylation state in the regulatory element regions of HPA axis-related genes (such as NR3C1), and this epigenetic write-in is activated when it suffers a second blow in adulthood, significantly increasing the risk of depression and post-traumatic stress disorder. In this process, histone modifications, including acetylation, crotonylation and methylation, serve as critical pathogenic targets and mechanisms for the onset of psychiatric disorders in adulthood.^[152,154]^

## Discussion

**Figure 3:**
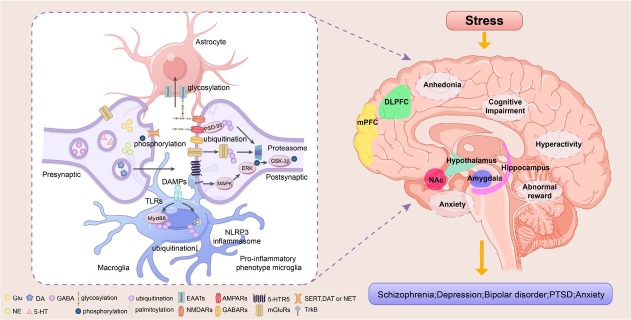
PTMs targets in the brain: neurotransmitters, neurocircuits and neuroinflammation. In the brain, post-translational modifications (PTMs) of proteins significantly influence metabolic and molecular pathways, which subsequently affect neurotransmitter systems and behavioral regulation circuits, especially those related to anhedonia, avoidance, and hyperarousal, commonly observed in various neuropsychiatric disorders, including schizophrenia, depression, *etc*. At the molecular level, pro-inflammatory cytokines induced by activated microglia can increase the expression and function of presynaptic reuptake transporters for serotonin (5-HT), dopamine (DA), and norepinephrine (NE), thereby reducing the availability of these monoamine neurotransmitters. Also, activated microglia can convert kynurenine to quinolinic acid (QUIN), which binds to N-methyl-D-aspartate receptors (NMDARs) and α-Amino-3-hydroxy-5-methyl-4-isoxazolepropionic acid receptor (AMPARs), and impair the release and reuptake of glutamate (Glu) system. PTMs such as phosphorylation, glycosylation, palmitoylation, and ubiquitination play crucial roles in regulating neurotransmitter receptor function, neuronal signaling, and inflammatory responses. Abnormalities in these modifications induced by stress may contribute to several aspects of behavior, such as reward motivation and anhedonia in circuits involving the basal ganglia, medial prefrontal cortex (mPFC), Nucleus Accumbens (NAc) and subgenual and dorsal anterior cingulate cortex (sgACC and dACC, respectively), while also activating circuits regulating anxiety, arousal, alarm and cognition including the amygdala, hippocampus, arcuate nucleus and insula. PTSD: post-traumatic stress disorder.

In recent years, despite a deeper understanding of the mechanisms underlying mental disorders, traditional research has primarily focused on gene mutations and neurotransmitter imbalances, neglecting the potential critical role of PTMs in the development of these diseases. PTMs not only regulate signal transduction but also serve as “memory carriers” of the pathological states associated with mental disorders. By creating a persistent PTM profile within individuals’ neurons, these modifications can reflect the history of early environmental factors or psychological stress, influencing adulthood response patterns to subsequent chronic stress, further leading to psychiatric disorders (Figure 3).

We highlight the dynamic nature of PTMs and their importance in mental health, suggesting they provide a novel perspective on the complexity of mental disorders. Advancements in PTMs research present promising avenues for understanding treatment-resistant psychiatric conditions, influenced by intricate factors such as disease mechanisms and diagnostic challenges. We specifically address PTMs’ impact on comorbidities associated with treatment-resistant disorders, noting the heterogeneity of symptoms, including psychotic features and cognitive impairments, in affected patients.

### Current therapeutics and emerging strategies

The prescription logic of traditional psychotropic drugs relies on the classic framework of synaptic transmission regulation, and its target distribution is as follows: about one-third of the drugs target G protein-coupled receptors (such as dopamine D2 receptors, 5-HT_1A_ receptors) and mediate slow nerve signal transmission by regulating the second messenger system; one-third act on neurotransmitter transporters (such as SERT and DAT), increasing neurotransmitter concentration in the synaptic gap by blocking reuptake; the remaining one-third interferes with voltage-gated ion channels (such as calcium channels, potassium channels) or key metabolic enzymes (such as monoamine oxidase MAO), achieving rapid modulation of neuronal excitability.^[155]^ However, the grim reality revealed by clinical empirical research is that 30%–50% of patients fail to achieve clinical remission even if they receive sufficient standard treatment.^[48,84]^ The treatment-resistant phenomenon constitutes the core bottleneck in the current field of psychopharmacology.

In order to solve the problem of individual response differences in the early days, the research paradigm focused on the direct effect hypothesis of genetic polymorphisms. For example, at the level of drug metabolism, loss-of-function alleles of cytochrome P450 enzymes can cause abnormal fluctuations in blood drug concentrations.^[156]^ Although these findings partially explain pharmacokinetic variation, their predictive power is still limited to a certain therapeutic effect, implying that there is still a huge translational gap between individual genetic factors and clinical phenotypes.

With the deepening of research on PTMs, the molecular nature of this boundary has been gradually revealed. The path from genetic polymorphisms to protein function is not linearly conducted, but is reshaped by a highly dynamic and context-dependent PTMs network. Taking the DISC1 gene as an example, the impairment of *DISC1* locus may not affect the expression of the protein itself, but its dual-site phosphorylation of Ser58 and Ser713 can lead to incorrect polarization of cortical neurons, while GSK3β inhibitors can effectively rescue abnormal axon initial segments and the corpus callosum.^[157]^ In addition, circuit abnormalities caused by *DISC1* site defects may also involve limited transport of neurotrophic factors such as BDNF. In this process, reducing the phosphorylation of Huntingtin serine-421 through lithium can help alleviate the situation.^[158]^ More importantly, PTMs constitute the molecular intersection of environmental stress and genetic susceptibility. The increase in glucocorticoids induced by chronic stress can directly activate histone acetyltransferases, inscribing epigenetic marks into HSP90 within 10-day stress, and the intensity of its effect far exceeds the lifetime contribution of static genetic polymorphisms.^[128]^

Mouse models with genetic mutations related to mental disorders display behavioral changes across diverse domains, consistently exhibiting abnormalities in PTMs associated with neurotransmission and synaptic development. Targeting these abnormal PTMs may be vital for addressing the genetic underpinnings of treatment-resistant psychiatric disorders. Notably, both classic antipsychotics and emerging treatments like ketamine influence abnormal PTMs, indicating a potential therapeutic strategy.^[159,160]^ Besides, the efficacy of traditional psychotropic drugs such as lithium and classic antidepressants may act on their rebalancing effect on pathological PTMs. By inhibiting the activity of protein kinase Akt1, lithium not only blocks the phosphorylation modification of substrates by this kinase,^[158]^ but also indirectly increases the level of histone acetylation, thus reversing the stress-induced suppression of BDNF gene transcription.^[161]^ HDAC inhibitors, as emerging antidepressant drug candidates, can directly reshape H3K9ac modification patterns in the prefrontal cortex and restore synaptic plasticity gene expression profiles.^[162]^ The non-classical PTMs regulatory effects of these classic drugs and emerging therapeutics not only explain the molecular basis of their broad-spectrum efficacy,^[163]^ but also suggest that future drug development should go beyond the receptor occupancy theory and turn to epigenetic regulators or kinase inhibitors that can repair pathological PTMs networks, providing a way to solve the problem of treatment resistance.

### Biomarker potential from diagnosis to prognosis

In the dimensions of diagnostic stratification and prognostic assessment for mental disorders, the profile of PTMs is increasingly highlighting its clinical translational value as a dynamic molecular fingerprint. Unlike static genetic polymorphisms, PTMs can capture real-time abnormalities in the activation or inactivation of signaling pathways during disease activity, providing a higher temporal and spatial resolution biological basis for clinical decision-making. Specifically, in terms of enhancing diagnostic efficacy, previous studies have found that patients with schizophrenia exhibit significantly elevated levels of polysialic acid (PSA) in their peripheral serum, while no similar changes have been observed in the blood of patients with depression or bipolar disorder.^[164]^ This disease-specific glycosylation phenotype offers a potential molecular biomarker for the differential diagnosis of psychiatric disorders. The hyperphosphorylation of specific tau protein sites in CSF (such as p-tau181 and p-tau217) not only aids in differentiating AD with psychiatric symptoms but also reveals the risk of tau-related psychiatric disorders and antipsychotic side-effects.

More revolutionary is that modification omics signatures can capture molecular changes in the subclinical stage. For example, in patients who respond to fluvoxamine, SERT is marked for degradation through polyubiquitination, allowing it to be recognized and degraded by the proteasome, thus maintaining a dynamic balance in the clearance of 5-HT from the synaptic cleft. However, in lymphoblasts derived from treatment-resistant patients, the recognition capacity of E3 ubiquitin ligases for SERT is impaired, leading to decrease in SERT ubiquitination and consequently slowing down the proteasomal degradation.^[164]^ From this perspective, reversing ubiquitination defects may provide precise intervention targets to overcome drug resistance.

However, whether the peripheral blood modification spectrum can truly reflect central pathology is still controversial. Meanwhile, the reliability of research findings related to PTMs in clinical translation still faces significant constraints, with the fundamental bottleneck being the heterogeneity of study designs. Cross-sectional studies of disease stages fail to capture the dynamic alterations of PTMs. Additionally, the lack of standardized comparability in cross-matrix comparisons of sample sources, ranging from peripheral blood mononuclear cells, CSF, to postmortem brain tissue, coupled with confounding factors such as treatment history, comorbid diagnoses, and genetic backgrounds, limits the ecological validity of existing evidence. This makes it difficult to establish robust peripheral-central correlations and causal inferences in clinical practice.

### Study strategies and advanced technologies

As research into PTMs in neuropsychiatric disorders progresses, there is growing optimism that these insights will lead to reliable treatments that address the underlying molecular dysfunctions in these complex conditions. Multiomics studies,^[165]^ including genomics, transcriptomics, proteomics, and metabolomics, are the primary ways to address barriers between clinical and animal models. The base editing screens system centered on PTMs has achieved genome-wide functional mapping of phosphorylation sites through deep integration with high-throughput phenotypic screening strategies.^[166]^ Meanwhile, the development of single-cell multi-omics technologies has made it possible to simultaneously detect multiple modifications, such as phosphorylation, acetylation, and ubiquitination, in individual neurons.^[167]^ This breakthrough provides a perspective on cellular heterogeneity for elucidating the crosstalk network of PTMs. Even though, the ultimate effects of PTM signals are not executed by the modifications themselves but rely on dynamic recognition and recruitment by specific reader proteins. These modular domains serve as molecular interfaces in signal transduction. To address this bottleneck, the recently developed multifunctional amino acid probe ADdis-Cys forms covalent bonds with adjacent proteins and combined with mass spectrometry technology, achieving multiple functional capture.^[168]^ By integrating these multifaceted data and validating the evidence of tissue cues in clinical patients in different species models, a more comprehensive understanding about the molecular mechanisms of psychiatric illnesses will be possible.

A key area of research focuses on developing personalized medical approaches targeting PTMs pathways. Given individual variability in PTMs influenced by genetic and environmental factors, tailored interventions can address specific PTMs dysregulations, enhancing treatment efficacy for mental disorders. As illustrated in Table 4 of the literature review, PTMs changes across various brain regions and cell types in response to stress paradigms show significant variability, with certain patterns linked to behavioral outcomes. Different PTMs are regulated by distinct enzymes, and current strategies often involve modulating these enzymes through techniques such as knockdown or inhibition in animal models. Furthermore, some Chinese medicine extracts demonstrate protective effects against aberrant PTMs, although the universal mechanisms underlying these effects require further exploration. Emerging technologies like CRISPR-based gene editing and single-cell proteomics offer opportunities to elucidate the functional impacts of specific PTMs within neuronal populations. These methodologies can deepen our understanding of PTMs signaling in the brain and help identify new therapeutic targets for psychiatric disorders, paving the way for precision medicine in neuroscience.

## Conclusion

In conclusion, understanding PTMs in the brain is at a critical juncture, with current insights poised to lead to breakthroughs in neuroscience that could transform the prevention, diagnosis, and treatment of psychiatric disorders. Continued investment in this field is essential for unlocking these possibilities and improving patient outcomes.
